# METTL16 in human diseases: What should we do next?

**DOI:** 10.1515/med-2023-0856

**Published:** 2023-11-30

**Authors:** Hui Zhang, Mengqi Yin, Hua Huang, Gongfang Zhao, Mingliang Lu

**Affiliations:** Department of Gastroenterology, Wuhan Tongji Aerospace City Hospital, Wuhan, Hubei Province, 430000, China; Department of Gastroenterology, The Second Affiliated Hospital, Kunming Medical University, Kunming, 665000, Yunnan Province, China; Department of Neurology, Wuhan No. 1 Hospital, Wuhan, Hubei Province, 430000, China; Department of Gastroenterology, Beijing Luhe Hospital, Capital Medical University, Beijing, 101149, PR China

**Keywords:** METTL16, m6A methyltransferase, human diseases, RNAs, cancers

## Abstract

METTL16 is a class-I methyltransferase that is responsible for depositing a vertebrate-conserved S-adenosylmethionine site. Since 2017, there has been a growing body of research focused on METTL16, particularly in the field of structural studies. However, the role of METTL16 in cell biogenesis and human diseases has not been extensively studied, with limited understanding of its function in disease pathology. Recent studies have highlighted the complex and sometimes contradictory role that METTL16 plays in various diseases. In this work, we aim to provide a comprehensive summary of the current research on METTL16 in human diseases.

## 1 Introduction

m6A modification is considered the most prevalent RNA modification in mammalian cells, emerging in both coding RNAs and non-coding RNAs [1–5]. This epi-transcriptional modification is known to promote the initiation and progression of many human diseases [6–8]. As is widely understood, m6A modification is regulated by writers, readers, and erasers, and exerts its effects by influencing RNA splicing, stability, transcription, translation, and decay [8–13]. To date, many m6A writers, such as METTL3, METTL14, WTAP, KIAA1429, and RBM15, have been thoroughly investigated, while the role of METTL16 in this process remains poorly understood.

METTL16 belongs to the class-I methyltransferase family, which has a vertebrate conserved S-adenosylmethionine (SAM) site [14–16]. Structural studies over the past few decades have confirmed that METTL16 consists of 562 amino acids, involving to seven beta-strands in the Rossmann fold. The quaternary structure of METTL16 is formed by an N-terminal methyltransferase domain (MTD) and two C-terminal vertebrate-conserved regions (VCRs), the MTD and the two VCRs are flanked by two disordered regions [14,17,18]. Accumulated studies have shown that METTL16 can exist as both a homodimer and a monomer [17,19], and is found in both the cytoplasm and nucleus [15]. METTL16 directly binds to target RNA that possesses a specific sequence and stem-loop structure, and also binds directly to the translation initiation complex (TIC) to regulate translation [5,17,20–22]. Studies have shown that METTL16 is closely related to almost all types of RNAs, as well as many RNA regulators and effectors [23–25]. Other studies have determined that METTL16 is also involved in maintaining SAM homeostasis [17,20]. Particularly, METTL16 can directly interact with ribosomal RNA to enhance translation, which distinguishes it from other METTL members such as METTL3 [22].

**Figure 1:**
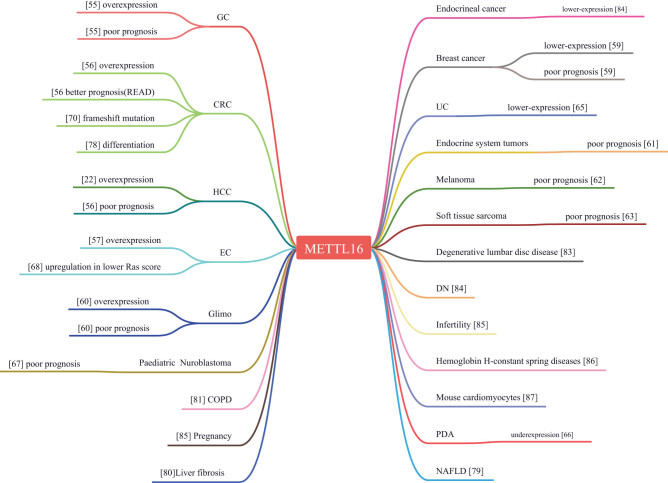
Schema of METTL16 in diseases.

Numerous studies have highlighted the significance of METTL16 in various cellular functions and human diseases, though investigations have been relatively limited in past decades. In this work, we have summarized the current achievements in METTL16 studies related to human diseases, as illustrated in Figure 1. We anticipate that METTL16 will be a key area of focus in future studies on human diseases.

## 2 N6-methyladenosine modification

The m6A modification, formally known as N6-methyladenosine modification, represents a prominent type of RNA chemical modification, involving methylation at the N6 position of adenosine and standing as the most prevalent internal RNA modification detected within eukaryotic messenger RNAs (mRNAs), long non-coding RNAs (lncRNAs), and diverse other RNA species [26–29]. The intricate orchestration of writers, erasers, and readers collaboratively governs the dynamic addition and removal of this modification [27]. Its influence extends across various realms of RNA biology, spanning splicing [30], stability [31], translation [32], and localization processes [33]. Furthermore, the m6A modification plays a pivotal role in a myriad of physiological processes, including embryonic development [34], cell differentiation [35], fertility [36], metabolism [37], and stress responses [38]. Crucially, the dysregulation of m6A modification emerges as a key player in a spectrum of human diseases, notably encompassing cancer [8], neurodegenerative disorders [39], and metabolic anomalies [40].

### 2.1 m6A writers, erasers, and readers

The m6A writers, erasers, and readers constitute the molecular composition of m6A RNA methylation regulator proteins [41]. These proteins collectively shape the m6A modification landscape on mRNAs and noncoding RNAs. Writers are responsible for inserting m6A marks, erasers remove them, and readers recognize and interpret the modification [42]. The primary writers category includes the METTL3–METTL14–WTAP methyltransferase complex, which orchestrates the addition of m6A modifications to specific adenosine residues in RNA molecules; while METTL3 and METTL14 act as the core catalytic units, WTAP enhances stability [6,18,40]. Erasers, represented by FTO and ALKBH5 demethylases, play a pivotal role in the reversible nature of m6A marks, influencing RNA stability and translation. FTO and ALKBH5 exhibit distinct substrate preferences and functions that influence m6A dynamics across different cellular contexts [43,44]. Readers, comprising diverse proteins like YTH domain-containing proteins (YTHDC1, YTHDC2, and YTHDF1-3) and other m6A-binding proteins (IGF2BP1-3 and HNRNPA2B1), interpret the m6A modification, triggering cellular responses that impact mRNA processing, splicing, and translation [45–48]. These intricate interactions contribute to elaborate regulatory networks controlling gene expression and cell fate determination. The interplay among writers, erasers, and readers orchestrates a finely tuned regulatory network governing the dynamic m6A modification landscape. This collaboration integrates multiple layers of regulation, allowing cells to adapt to environmental cues and developmental signals. Maintaining the balanced function of m6A writers, erasers, and readers is crucial, as dysregulation has been linked to various diseases. Comprehensive reviews for detailed discussions on the role of m6A regulatory proteins in human diseases are already available in literature, as well as target therapy [42,49–53]. For comprehensive information on current m6A methyltransferases, refer to Table 1.

**Table 1:** m6A writers, erasers, and readers

Types	m6A regulator	Full names
**Writers**		
	METTL3	Methyltransferase -like 3
	WTAP	Wilms tumor 1-associated protein
	METTL14	Methyltransferase -like 14
	VTRMA [KIAA1429]	Vir-like ni6A methyltransferase associated
	RBM15	RNA binding motif protein 15
	RBM15B	RNA binding motif protein 15B
	METTL16	Methyltransferase-like 16
	ZC3H13	Zine finger CCCH-type containing 13
	METTL5	Methyltransferase-like 5
	ZCCHC4	Zine finger CCHC-type containing 4
	METTL4	Methyltransferase-like 4
	MT-A70	S-adenosylmethionine -binding subunit of human mRNA
	METTL7A	Methyltransferase like 7A
	METTL7B	Methyltransferase like 7B
	METTL11A	Methyltransferase-like protein 11A
	NSun2	Catalyses m6A modification
**Erasers**		
	FTO	Fat mass and obesity associated protein
	ALKBH5	AlkB homolog 5
	ALKBH3	AlkE homolog 3
**Readers**		
	YTHDF2	YTH N6-methyladenosine RNA binding protein 2
	YTHDF1	YTH N6-methyladenosine RNA binding protein 1
	elF3	Eukaryotic translation initiation factor 3 subunit A
	HNRNFA2B1	Heterogeneous nuclear ribonucleoprotein A2/B1 nucleus
	HNRNPG	Heterogeneous nuclear ribonucleoprotein G
	YTHDC1	YTH domain containing 1
	YTHDF3	YTH N6-methyladenosine RNA binding protein 3
	YTHDC2	YTH domain containing 2
	IGF2BP1	Insulin-like growth factor 2 mRNA binding protein 1
	IGF2BP2	Insulin-like growth factor 2 mRNA binding protein 2
	IGF2BP3	Insulin-like growth factor 2 mRNA binding protein 3
	FMRP	Fragile X mental retardation protein
	PRRC2A	Proline rich coiled-coil 2 A
	RBM33	RNA-binding motif protein 33
	NKAP	Mediates pri-miRNAs processing
	RBM45	RNA-binding motif protein 45
	HNRNPC	Heterogeneous nuclear ribonucleoprotein C

## 3 METTL16

**Figure 2:**
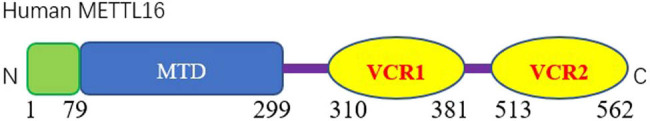
Sketch of human METTL16.

More recently, another m6A methyltransferase, METTL16, has been identified. Although the function of this enzyme is not fully understood, its structure has been thoroughly studied. Human METTL16, belonging to class I SAM-MTases, possess a conserved SAM locus found in vertebrates and are made up of 562 amino acid residues [14–16]. The construction primarily consists of an N-terminal MTD and two VCRs located at the C-terminus. Two disordered districts are found on either side of MTD and the two VCRs, as shown in Figure 2 [14,17,54]. As per current understanding, METTL16 is found in both the nucleus [42] and cytoplasm [43] and typically exists as a monomer [44,45]. However, upon binding with its substrate, MALAT1 triple-helix RNA, it forms a homodimer [16,54].

### 3.1 METTL16 in cancers

m6A modification and its associated regulated factors, including METTL3/METTL14, YTHDC, FTO, and ALKBH5, etc., have been widely researched for their roles in various types of cancer. However, the investigation of METTL16 in cancer is limited and there are only a few reports available to date.

### 3.2 Expression and prognostic significance of METTL16 in cancer

METTL16 has been identified as a potential gene involved in the initiation and progression of cancer. However, its expression varies across different tumor types and is associated with different outcomes. In some cases, it has been identified as highly expressed and associated with poor outcomes. For example, Wang et al. reported that METTL16 is highly expressed in gastric cancer and predicts worse survival in patients. The underlying molecular mechanism mainly involves METTL16 functioning as an m6A methyltransferase to promote cancer cell proliferation [55]. A clinical cohort study containing 66 hepatocellular carcinoma (HCC) tissues and 21 adjacent normal tissues determined that higher METTL16 expression group displayed a worse clinical outcome [56]. Additionally, bioinformatics analyses have shown that METTL16 is overexpressed in esophageal cancer [57], colorectal cancer (CRC) [58], and predicts poor survival in HCC, CRC, endocrine system tumors, glioma, melanoma, soft-tissue sarcomas, and breast cancer [22,58–63]. While in other cases, there also have been reported that METTL16 is underexpressed in endometrial cancer [64], urothelial carcinoma [65], pancreatic ductal adenocarcinoma [66], and breast cancer [59]. In patients with pediatric neuroblastoma, Zhang et al. found that METTL16 could affect the overall survival and disease-free survival of patients [67]. In addition, in a RAS-related gene score of esophageal squamous cell carcinoma, METTL16 was found upregulated in the lower score group and predicted a better prognosis [68]. In a study to investigate m6A associated genes between patients with TP53 wild-type and mutation groups, METTL16 exhibited divergent expression between the groups [69].

METTL16 showed divergent expression in cancers, even in the same cancer, it has been found in different expressions in different studies, the underlying reasons should be further studied. Usually, METTL16 leads to bad prognosis; however, the effects on cancer prognosis can be modulated by other genes. These findings suggest that its role in cancer is complex and context-dependent. Recent studies uncovered that METTL16 can function as both m6A-dependent way or non-dependent way, both as a writer and a reader, both in cytoplasm or in nucleus, both regulate splice or translation, all of which showed a fantastic of METTL16. However, limited studies in specific cancers were conducted. Further research is needed to elucidate the molecular mechanisms underlying these effects and to explore the potential of METTL16 as a therapeutic target for cancer treatment.

### 3.3 METTL16 gene mutation in cancer

The identification of mutations in genes that diverged may play a significant role in understanding individual differences and recognizing diverse clinicopathological characteristics. Various studies have revealed that the METTL16 gene mutation is widespread in several types of cancer. In high microsatellite instability colorectal cancers, METTL16 contains frameshift mutations that are not present in normal tissues [70]. An analysis of METTL16 copy number variations (CNVs) using bioinformatics has shown that CNVs of this gene are common in cancers and can influence gene expression, leading to a worse prognosis. In more than 60% of sarcoma patients and 62.16% of HCC samples, METTL16 CNVs were present [63]. Furthermore, anti-tumor drugs have been associated with METTL16 mutation, and a study on methotrexate revealed that drug sensitivity to melanoma was linked to METTL16 mutations [62]. However, experimental validation and clinical data were lacking, indicating the need for further research in this area. Overall, the current study of METTL16 gene mutation showed the importance of gene mutations in cancers and the need for further research to develop effective treatment options for patients with cancer.

### 3.4 METTL16 and lncRNA

The lncRNA MALAT1 has an ENE+A structure and an METTL16 recognition sequence, allowing it to interact directly with METTL16 [24]. Studies have shown that MALAT1 can both facilitate and suppress cancer development and is common in various cancers [71,72]. An analysis of 89 pathways in 64 different cancers has revealed the presence of MALAT1 [73]. Additionally, MALAT1 can interact with microRNA and cancer drugs [74,75]. These studies highlights the significant associations of lncRNA MALAT1 or microRNA with METTL16, which has been shown to play a crucial role in regulating cellular signals and promoting cancer development. Another lncRNA, named lncRNA RAB11B-AS1, has recently been found to directly bind to METTL16, promoting cancer development in an m6A-dependent manner by decreasing lncRNA stability [76]. These findings suggest that METTL16 interacts with lncRNA and miRNA to regulate cellular signals and contribute to cancer development. However, further experiments are necessary to fully understand the role of METTL16 and RNAs in cancer.

### 3.5 METTL16 and DNA damage response (DDR)

DDR has been linked to the development of tumors. Studies have shown that during the early stages of DDR, there is a significant increase in N6-adenosine methylation in RNA following UV-micro-irradiation. In later stages, small RNAs, including snRNAs and snoRNAs, in the vicinity of DNA lesions were found to be methylated, and it was determined that METTL16 is the sole methyltransferase responsible for this process [77]. These findings suggest that METTL16 may play a critical role in cancer development related to DDR. However, the precise mechanism by which METTL16 contributes to DDR remains unknown.

### 3.6 Others

METTL16 has been reported to affect cell differentiation and protein translation. Studies have shown that the level of METTL16 is negatively associated with tumor cell differentiation and that the m6A level decreases with better differentiation [78]. METTL16 can regulate protein translation and aggravate cancer development by directly binding to TIC [22].

### 3.7 METTL16 in other diseases

Recent studies have shed light on the potential involvement of METTL16 in non-cancerous diseases across various human systems. In the digestive system, in HFD-induced mice and cell NAFLD models, the expression levels of METTL16 were substantially increased in the NAFLD model *in vivo* and *in vitro*. Furthermore, mechanism studies showed that METTL16 upregulated the expression level of lipogenic genes CIDEA in HepG2 cells [79]; otherwise, METTL16 also plays vital role in liver fibrosis [80]. In the respiratory system, research has shown that exposure to PM2.5 induces pulmonary vessel damage in an m6A-dependent manner through METTL16, providing new insights into the mechanisms of chronic obstructive pulmonary disease and cancer [81]. Additionally, in a mouse model of acute respiratory distress syndrome induced by LPS, researchers observed a gradual increase in m6A levels over 6 h, followed by a decrease. During this process, the METTL16 protein increased consecutively, while METTL16 mRNA decreased after LPS induction. The differences between METTL16 mRNA and protein expression warrant further investigation [82]. In the spinal system, studies have shown that the expression of METTL16 differs between human degenerative nucleus pulposus and control groups, and METTL16/MAT2A axis aggravates apoptosis of nucleus pulposus cells by regulating splicing, maturation, and degradation of MAT2A pre-mRNA [83]. In the endocrine system, a cross-sectional study for people of Middle Eastern descent revealed an association between METTL16 and diabetic nephropathy [84]. Additionally, in the reproductive system, research found that although the m6A level of pregnancy was elevated consistently, METTL16 was lower in patients with infertility and recrudescence abortion [85]. Finally, studies suggest that METTL16 may also play a role in cardiovascular and hematological systems, with low expression in immature RBCs of Hb CS thalassemia versus healthy controls [86] and contributing to mouse cardiomyocytes [87]. Moreover, METTL16 was identified to play a role in erythropoiesis through the repair of DDR [88]. Overall, these findings highlight the potential involvement of METTL16 in various non-cancerous diseases, emphasizing the wide roles of METTL16 in human disease, indicating the need for further research to elucidate the mechanisms of action and potential therapeutic applications.

## 4 Discussion and conclusion

METTL16 plays a vital role in cellular biogenesis and is associated with various human diseases. Studies have found that METTL16 knockdown can cause a significant decrease in the installation of m6A/A [17], and can even lead to embryonic lethality in mice [19]. While there is significant evidence that METTL16 is involved in both cancer and non-cancer diseases, the role that it plays is complex, with numerous paradoxes and contradictions. For example, METTL16 can be located in both the cytoplasm and nucleus, can function as both a writer and a reader, and can have both higher and lower levels of expression even in one disease of two studies. Additionally, it can exert its effects in multiple ways, such as directly binding to TIC or regulating target RNA splicing, stability, and more. It can function as both a promoter and as a suppressor of diseases, making it challenging to determine its overall effect. However, the studies of mechanisms or function of METTL16 in human diseases is absent, Moving forward, further research is needed to answer critical questions, such as how METTL16 influences human diseases, the mechanisms by which it exerts its effects, and whether its role varies depending on the cell type, disease, or individual.

Recognizing the pivotal role of METTL16 in disease contexts is of utmost importance. So, what is our next course of action? Recent research has unveiled a promising treatment approach for PDAC, particularly in cases where METTL16 expression is heightened, by combining PARPi with gemcitabine [89]. This breakthrough emphasizes the potential of targeting METTL16 for cancer therapy. Furthermore, it is crucial to evaluate METTL16 and its downstream RNA methylation targets as potential markers for disease diagnosis, prognosis, and monitoring of disease progression. Consequently, there is a pressing need to develop assays and diagnostic tools based on these findings. Simultaneously, we are actively exploring animal models and conducting clinical trials to deepen our comprehension. Additionally, we should embark on functional genomic studies aimed at uncovering the genes and pathways influenced by METTL16-mediated RNA methylation, shedding light on the broader implications of METTL16 in disease biology. Another avenue worth investigating is patient stratification, where we can assess METTL16-related mechanisms for their ability to categorize patients into subgroups with distinct disease outcomes or treatment responses. This approach holds significant promise for advancing personalized medicine initiatives.

## Abbreviations

CNV: copy number variations
CRC: colorectal cancer
ENE+A: element for nuclear expression with a downstream A-rich tract
HCC: hepatocellular carcinoma
RBC: red blood cell
